# N-acetyl-cysteine inhibits liver oxidative stress markers in BALB/c mice infected with Leishmania amazonensis

**DOI:** 10.1590/0074-02760160403

**Published:** 2017-02

**Authors:** Juciano Gasparotto, Alice Kunzler, Mario Roberto Senger, Celeste da Silva Freitas de Souza, Salvatore Giovanni de Simone, Rafael Calixto Bortolin, Nauana Somensi, Felipe Dal-Pizzol, José Claudio Fonseca Moreira, Ana Lúcia Abreu-Silva, Kátia da Silva Calabrese, Floriano Paes Silva, Daniel Pens Gelain

**Affiliations:** 1 Universidade Federal do Rio Grande do Sul, Instituto de Ciências Básicas da Saúde, Departamento de Bioquímica, Centro de Estudos em Estresse Oxidativo, Porto Alegre, RS, Brasil; 2 Fundação Oswaldo Cruz-Fiocruz, Instituto Oswaldo Cruz, Laboratório de Bioquímica Experimental e Computacional de Fármacos, Rio de Janeiro, RJ, Brasil; 3 Fundação Oswaldo Cruz-Fiocruz, Instituto Oswaldo Cruz, Laboratório de Imunomodulação e Protozoologia, Rio de Janeiro, RJ, Brasil; 4 Fundação Oswaldo Cruz-Fiocruz, Instituto Oswaldo Cruz, Centro de Desenvolvimento Tecnológico em Saúde, Rio de Janeiro, RJ, Brasil; 5 Universidade do Extremo Sul Catarinense, Programa de Pós-Graduação em Ciências da Saúde, Laboratório de Fisiopatologia Experimental, Criciúma, SC, Brasil; 6 Universidade Estadual do Maranhão, Departamento de Patologia, São Luís, MA, Brasil

**Keywords:** Leishmania amazonensis, antioxidant treatment, liver damage

## Abstract

**BACKGROUND:**

Leishmaniasis is a parasitosis caused by several species of the genus *Leishmania*. These parasites present high resistance against oxidative stress generated by inflammatory cells.

**OBJECTIVES:**

To investigate oxidative stress and molecular inflammatory markers in BALB/c mice infected with *L. amazonensis* and the effect of antioxidant treatment on these parameters.

**METHODS:**

Four months after infection, oxidative and inflammatory parameters of liver, kidneys, spleen, heart and lungs from BALB/c mice were assessed.

**FINDINGS:**

In liver, *L. amazonensis* caused thiol oxidation and nitrotyrosine formation; SOD activity and SOD2 protein content were increased while SOD1 protein content decreased. The content of the cytokines IL-1β, IL-6, TNF-α, and the receptor of advanced glycation endproducts (RAGE) increased in liver. Treatment with the antioxidant *N*-acetyl-cysteine (20 mg/kg b.w) for five days inhibited oxidative stress parameters.

**MAIN CONCLUSIONS:**

*L. amazonensis* induces significant alterations in the redox status of liver but not in other organs. Acute antioxidant treatment alleviates oxidative stress in liver, but it had no effect on pro-inflammatory markers. These results indicate that the pathobiology of leishmaniasis is not restricted to the cutaneous manifestations and open perspectives for the development of new therapeutic approaches to the disease, especially for liver function.

Leishmaniasis is a parasitosis caused by several species of the genus *Leishmania*, an obligate intramacrophagic parasite (Alvar et al. 2012). It is estimated that 350 million people are under risk of infection, while 12 million people are currently infected (Desjeux 2004) and the annual incidence is 0.7 to 1.2 million new cases. *Leishmania amazonensis* causes localised and diffuse cutaneous lesions and is associated to the dissemination of cutaneous leishmaniasis.

The immune response induced by *Leishmania* infection, as well as the mechanisms of host resistance and pathogenesis of disease, is not completely understood. Reactive nitrogen and oxygen species are produced during oxidative burst by activated macrophages. Despite the fact that many species are resistant to oxidative stress, macrophages activation has been suggested to be involved in the outcome of *Leishmania* infection (Bisti et al. 2006). These characteristics probably evolved as a parasite’s strategy of resistance against the oxidising agents generated by inflammatory cells during activation to destroy intracellular and extracellular pathogens.

Among the host responses against *Leishmania* infection, activation of NADPH oxidase, inducible nitric oxide synthase (iNOS) and myeloperoxidase may also contribute to tissue oxidative stress and damage (Blos et al. 2003). However, the inflammatory response may be further intensified depending on the parasite’s ability to cope with oxidative stress generated by the host. The intensification of oxidants production during this response may contribute to the development of the disease, as they further contribute to tissue injury. Oxidative stress and oxidative damage to biomolecules contribute to the progression of many diseases, particularly when the activation of pro-inflammatory responses is induced. This is observed not only in infectious diseases, but also in cardiovascular conditions, several types of cancer, neurodegenerative diseases and diabetes, which present pro-inflammatory components in the molecular mechanisms regulating their progression (Halliwell & Gutteridge 2007).

Thus, considering this relationship between pro-inflammatory activation and reactive species production, it is possible that oxidative stress is a contributing factor to tissue function impairment caused by leishmaniasis. We investigated how infection with *L. amazonensis* affected parameters of oxidative stress in different organs of BALB/c mice, in order to understand the contribution of oxidative stress to the pathogenesis and development of leishmaniasis. We observed that liver tissue presented altered oxidative stress parameters and that antioxidant treatment did not inhibit the effect of *L. amazonensis* infection on liver cytokine levels, although oxidative damage was inhibited. Our results indicate that liver oxidative stress does not occur prior to cytokine modulation, although chronic alterations in the oxidative status of this organ are known to contribute to the development of other complications at both tissue and systemic levels.

## MATERIALS AND METHODS

*Chemicals* - The chemicals used in the study were as follows: Glycine, H_2_O_2_ (hydrogen peroxide), catalase (CAT, EC 1.11.1.6), superoxide dismutase (SOD, EC 1.15.1.1), thiobarbituric acid, epinephrine, AAPH (2,2’-azobis[2-methylpropionamidine]dihydrochloride), trichloroacetic acid (TCA), 2,4-dinitrophenylhydrazine (DNPH), 5,5′-dithionitrobis 2-nitrobenzoic acid (DTNB), Bile salts, sodium dodecyl sulfate, DNP polyclonal antibody were purchased from Sigma-Aldrich^**®**^ (St. Louis, USA). Electrophoresis and immunoblot reagents were from Bio-Rad (Hercules, USA), GE Healthcare Brazilian Headquarter (São Paulo, Brazil) and Sigma-Aldrich^**®**^. RAGE polyclonal antibody, NFkB-p65 polyclonal antibody, SOD1 polyclonal antibody, β-actin polyclonal antibody and anti-rabbit immunoglobulin linked to peroxidase were from Cell Signalling technology^**®**^ (Beverly, USA). SOD2 monoclonal antibody, IL-1b polyclonal antibody, IL-10 monoclonal antibody, IL-6 polyclonal antibody and TNF-a polyclonal antibody were from Abcam^**®**^ (Cambridge, UK). ELISA microplates were from Greiner Bio-One (Monroe, USA) and ELISA TMB spectrophotometric detection kit was from BD Biosciences (San Diego, USA). Immunoblot chemiluminescence detection was carried out with the West Pico detection kit from Thermo Scientific Pierce Protein Biology Products (Rockford, USA). MilliQ-purified H_2_O was used for preparing solutions. All other reagents used in this study were of analytical or HPLC grade.

*Ethics statement* - All experimental procedures were performed in accordance with the National Institute of Health, Guide for the Care and Use of Laboratory Animals and the Brazilian Society for Neuroscience and Behavior recommendations for animal care. The experimental protocols were approved by the Oswaldo Cruz Foundation Committee of Ethics for the Use of Animals (CEUA-Fiocruz) protocol number P-36/11-3.

*Parasite strain and infection* - *L. amazonensis* (MHOM/BR/75/JOSEFA) were maintained by regular passage in BALB/c mice. Amastigotes were purified from the footpad lesions of mice. Female BALB/c mice (six weeks-old) obtained from Centro de Criação de Animais de Laboratório (CECAL - Fiocruz, Rio de Janeiro, RJ, Brazil) were infected subcutaneously in the right hind footpad with 10^5^ amastigotes. Five animals from two independent infections were used per group, a total of 80 animals were used to conduct all experiments. Antioxidant therapy of *N*-acetyl-cysteine (NAC) was applied for five days, consecutively, before euthanasia; a safe dose of 20 mg/kg per day was administered i.p., based in previous observations of toxicity assessment and antioxidant effectiveness. Four months after infection animals were euthanised by CO_2_ inhalation and liver, kidneys, spleen, heart and lungs were removed and oxidative and inflammatory parameters were assessed. To estimate parasite burden in the lesions, the entire infected footpads were removed and amastigotes were recovered from the lesions and counted, the parasite number range from 2.8 to 6.5 x10^8^.

*Oxidative stress parameters (catalase and superoxide dismutase activities)* - For oxidative stress assays was utilised phosphate buffer (PB) 50 mM (KH_2_PO_4_ and K_2_HPO_4_, pH-7.4) for homogenise and normalise the samples. Catalase (CAT; E.C. 1.11.1.6) activity was evaluated by following the rate of decrease in hydrogen peroxide (H_2_O_2_) absorbance in a spectrophotometer at 240 nm. Results are expressed as units of CAT/mg of protein. The activity of superoxide dismutase (SOD; EC 1.15.1.1) was measured by quantifying the inhibition of superoxide-dependent adrenaline auto-oxidation in a sample buffer; adrenochrome formation was monitored at 480 nm for 10 min (32ºC) in a spectrophotometer. Results are expressed as units of SOD/mg of protein.

*Oxidative damage to proteins (carbonyl)* - As an index of protein oxidative damage, the carbonyl groups were determined. The homogenate were divided into two aliquots of 300 mL (1 mg of protein). Proteins were precipitated by the addition of 150 mL of 20% TCA for 5 min on ice and centrifuged at 4.000 x *g* for 5 min. The pellet was dissolved with 100 mL of sodium hydroxide (NaOH) (200 mM) and 100 mL of hydrochloric acid (HCL) (2M) was added in blanks and DNPH (10 mM) was added to measure carbonyl groups. Samples were maintained for 30 min at room temperature. Proteins were precipitated with 20% TCA and washed three times with 500 mL of 1:1 ethanol:ethyl acetate with 15 min standing periods to remove the excess DNPH. Samples were dissolved in 200 mL of urea (8M) pH 2.3, and the absorbance was read at 370 nm.

*Sulfhydryl groups quantification* - Oxidative status of thiol groups was assessed by quantification of total reduced sulfhydryl (SH) groups in samples. Briefly, for total -SH content measurement, 60 mg sample aliquot was diluted in phosphate-buffered saline (PBS) (NaCl, Na_2_HPO_4_, KH_2_PO_4_), and 5,5′-dithionitrobis 2-nitrobenzoic acid (10 mM), and read in a spectrophotometer at 412 nm after 60 min of incubation in room temperature.

*Thiobarbituric acid reactive species (TBARS)* - As an index of lipid peroxidation, we quantified the formation of TBARS formed in an acid-heating reaction of samples with thiobarbituric acid. Briefly, the tissues homogenates were mixed with 0.6 mL of 10% trichloroacetic acid (TCA) and 0.5 mL of 0.67% thiobarbituric acid, and then heated in a boiling water bath for 25 min. TBARS were determined by the absorbance in a spectrophotometer at 532 nm. Results were normalised against the content of cell protein and expressed as TBARS/mg of protein.

*ELISA for detection of cytokines and nitrotyrosine* - IL-1β, IL-6, TNF-α, IL-10 and nitrotyrosine were quantified by indirect ELISA. Liver homogenate was placed in ELISA plate (40 mg/well). After 24 h incubation, plates were washed three times with Tween-Tris buffered saline (TTBS, 100 mM Tris - HCl, pH 7.5, containing 0.9 % NaCl, and 0.1 % Tween-20). Subsequently, 200 μL of primary antibody against IL-1β, IL-6, TNF-α, IL-10 or nitrotyrosine (1:1.000) were added and incubation was carried for 24 h at 4ºC. The plates were washed three times with TTBS and incubated with rabbit or mouse IgG peroxidase-linked secondary antibody (1:1.000) for 2 h. After washing the plate three times with TTBS, 200 μL of substrate solution (TMB spectrophotometric ELISA detection kit) were added to each well and incubated for 15 min. The reaction was terminated with 50 μL/well of 12 M sulfuric acid stopping reagent and the plate read at 450 nm in a microplate.

*Total reactive antioxidant potential (TRAP assay)* - The total reactive antioxidant potential (TRAP) was used as an index of non-enzymatic antioxidant capacity. This assay is based on the quenching of peroxyl radicals generated by AAPH by antioxidants present in a given sample. Briefly, a chemical system that generates peroxyl radicals at a constant rate (an AAPH-containing buffer) is coupled to a luminescent reactant (luminol) which emits photons proportionally to its oxidation. The samples were homogenised with glycine buffer (pH-8.6). The reaction was initiated by injecting luminol to the 0.1 M glycine buffer containing AAPH that resulted in steady luminescence emission. Equal amounts of samples are then added to this reaction system, and the luminescence emission at the moment following this addition (t = 0) is recorded. This initial emission reflects the production of free radicals by AAPH at the first moment right after sample addition and is related to the endogenous oxidant state of the sample. Following incubation, the thermal decomposition of AAPH produces luminescence at a constant rate (“system”), and the presence of free radical scavengers in the added sample will decrease this rate according to its content of non-enzymatic antioxidants. We followed TRAP luminescence emission for 80 min and calculated the area under the curve (AUC) relative to the system without samples (which was considered as 100% of luminescence emission at all time points. The addition of the homogenate samples decreases or facilitates the luminescence emission proportionally to its redox state. The luminescence emission was recorded in a MicroBeta^**®**^ luminescence counter (Perkin Elmer, USA).

*Western blot detection of dinitrophenyl (DNP)-labeled protein carbonyls* - Protein oxidation was assessed using western blots. Liver samples were homogenised with PBS (pH 7.4). The suspension was centrifuged at 10.000 × *g* for 20 min, supernatant was collected and protein concentration was assessed using Bradford assay. Briefly, 10 µL of 12% SDS was added to a 10 µL aliquot of sample containing 50 µg of protein. DNPH was prepared as a 10 mM solution in 10% trichloroacetic acid, added to the mixture (v/v) and incubated for 20 min at room temperature. The reaction mixture was neutralised and prepared for SDS-PAGE by adding 15 µL of 2 M Tris base containing 30% (v/v) glycerol. Liver samples (20 µg per well) were separated by SDS polyacrylamide gel electrophoresis (SDS-PAGE) and electro-blotted onto nitrocellulose membranes. More details for western blot assay are described in immunoblot section. Membranes were incubated overnight at 4ºC with rabbit polyclonal anti-DNP antibody at 1:5000 dilution range and then incubated with anti-rabbit IgG peroxidase-linked secondary antibody for an additional 1 h (1:5000 dilution range). All results were expressed as a relative ratio to the β-actin internal control.

*Immunoblot* - To perform immunoblot experiments, the tissue was homogenated with 1X RIPA buffer, centrifuged (10.000 *g* for 5 min at 4ºC) and the pellet proteins were measured by the Bradford method (Bradford 1976). Laemmli-sample buffer was added to complete volume according the protein content of each sample and equal amounts of cell protein (30 μg/well) were fractionated by SDS-PAGE and electro-blotted onto nitrocellulose membranes with Trans-Blot® SD Semi-Dry Electrophoretic Transfer Cell, Bio-Rad (Hercules, CA, USA). Protein loading and electro-blotting efficiency were verified through Ponceau S staining, and the membrane was washed with TTBS (Tris 100 mM, pH 7.5, 0.9% NaCl and 0.1% Tween-20). Membranes were incubated 20 min at room temperature in SNAP i.d.® 2.0 Protein Detection System Merck Millipore (Billerica, USA) with each primary antibody (anti-RAGE, anti-SOD1, anti-SOD2, anti-p65 and anti-β-actin) (1:500 dilution range) and after this period washed with TTBS. Anti-rabbit or mouse IgG peroxidase-linked secondary antibody was incubated with membranes for additional 20 min in SNAP (1:5000 dilution range), washed again and the immunoreactivity was detected by enhanced chemiluminescence using Supersignal West Pico Chemiluminescent kit from Thermo Scientific (Luminol/Enhancer and Stable Peroxide Buffer) in ImageQuant LAS 4000 (GE Healthcare). Densitometric analyses of western blots were performed with ImageJ software (ImageJ v1.49, National Institute of Health, USA). Blots were developed to be linear in the range used for densitometry. All results were expressed as a relative ratio to the β-actin.

*Statistical analysis* - Statistical analysis was performed with GraphPad Prism version 5.04 (GraphPad Software Inc., San Diego, USA). Student’s *t* test (one-tail) was applied for simple comparisons between organs of control and infected animals in experiments with only two groups. For experiments with multiple groups, one-way ANOVA was applied. Bonferroni’s post hoc test was used to evaluate efficacy of infection and Tukey’s post-hoc was applied to find significant differences among treatments considering all groups. The results of measurements were expressed as mean ± standard error of the mean (SEM). Differences were considered significant when p < 0.05.

## RESULTS

**Fig. 1:**
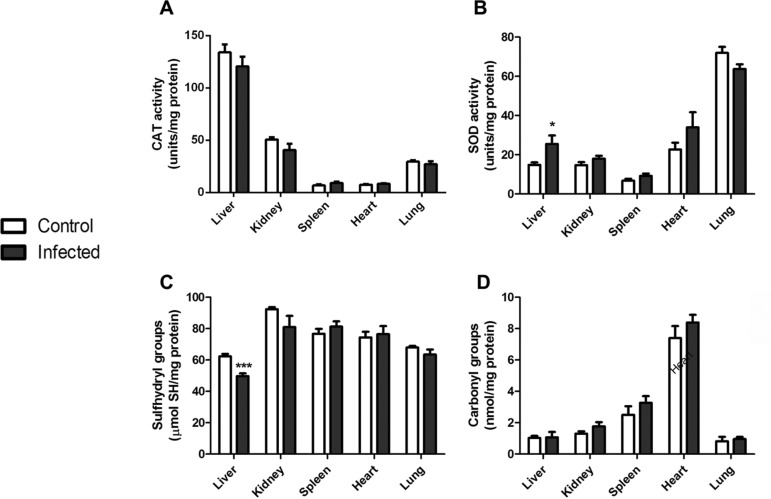
: oxidative stress parameters in organs of mice infected with *Leishmania amazonensis*. Liver, kidney, spleen, heart and lung tissues were isolated, homogenised and the activities of the antioxidant enzymes CAT (A) and SOD (B) were assessed. Free sulfhydryl (-SH) groups (C) were measured and protein carbonylation (D) was determined. Values represent mean ± standard error of the mean (SEM) of samples obtained from five mice of two distinct groups and two different infections. Student’s *t* test (two-tail) was applied for all data; * denotes p value < 0.05 and *** denotes p value < 0.0001.

To evaluate the host response induced by *L. amazonensis* at a systemic level, we assessed different parameters of oxidative stress in several organs of infected mice. We first compared the activities of the antioxidant enzymes CAT (Fig. 1A) and SOD (Fig. 1B) in the liver, kidney, spleen, heart and lung tissues from control and infected animals. No alterations were observed in tissues except for liver, where an increase in total SOD activity was evident (Fig. 1B). Oxidative modifications to proteins in these organs were also analysed and we observed that the content of sulfhydryl groups was decreased in the liver of infected animals (Fig. 1C), indicating a diminished content of reduced thiol groups; other organs were not affected, and protein carbonyl levels were not changed in any sample (Fig. 1D).

**Fig. 2:**
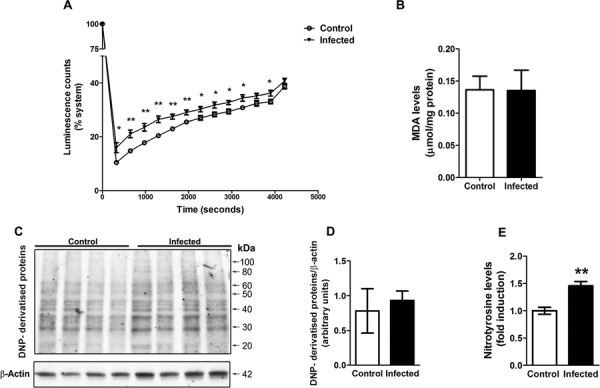
: liver parameters of oxidative stress in mice infected with *Leishmania amazonensis*. Total radical antioxidant parameter (TRAP) kinetic assay was employed with liver tissue samples (A). The luminescence emission was recorded during time; data points represent mean ± standard error of the mean (SEM) values of repeated measures. Lipoperoxidation was estimated by assessing TBARS levels (B). Western blot analysis for carbonyl groups was performed using an antibody against DNP (C, representative blot) and quantification relative to b-actin levels was performed (D). Detection of nitrotyrosine in samples was performed by ELISA (E). Values represent mean ± SEM of samples obtained from five mice of two distinct groups (two different infections). Student’s *t* test (two-tail) was applied for all data; * denotes p value < 0.05 and ** denotes p value < 0.005.

To better understand the effect of *L. amazonensis* infection on liver oxidative stress, we performed an assay to evaluate the non-enzymatic antioxidant capacity of this tissue by the TRAP parameter. In this assay, samples are subjected to a constant peroxyl production system and the kinetics of reactive species-derived luminescence in an enzyme-inactivating buffer is followed, thus determining the intrinsic capacity of the tissue to scavenge reactive species. We observed that liver samples from infected animals present a decreased capacity to inhibit AAPH-derived peroxyl production, indicating a decreased non-enzymatic antioxidant capacity of the tissue (Fig. 2A). On the other hand, the lipoperoxidation levels were not different between samples from control and infected animals (Fig. 2B). We also analysed protein oxidative damage by immunoassays against carbonyl and nitrotyrosine groups. No significant increases in DNP-derivatised proteins were detected by western blot analysis (Fig. 2C-D), confirming that carbonyl levels are not different between samples; however, nitrotyrosine levels were increased in the liver of infected animals (Fig. 2E).

**Fig. 3:**
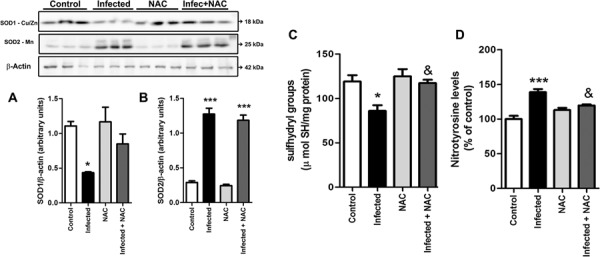
: effect of *N*-acetyl-cysteine (NAC) on parameters of liver oxidative stress. Animals infected with *Leishmania amazonensis* were subjected to five consecutive days of NAC (NAC, 20 mg/kg b.w.) i.p. injection before euthanasia and tissue collection. Western blot analyses were performed for SOD1 (A) and SOD2 (B) isoforms; b-actin was used as internal marker control. Free sulfhyfryl groups (C) and nitrotyrosine levels (D) were assessed as described earlier. Values represent mean ± standard error of the mean (SEM) of samples obtained from five mice of two distinct groups (two different infections). Data were analysed by ANOVA. Tukey’s multiple comparison was applied to detect significant differences between all groups; *p < 0.05 and ***p < 0.0001 compared to control group; &p < 0.05 compared to infected group.

Oxidative stress in infectious diseases is often related to the activation of inflammatory response, which may be triggered by the pathogenic agent and is often perpetuated due to the increased production of reactive species and consequent oxidative damage to biomolecules (di Penta et al. 2013). Thus, we subjected animals infected with *L. amazonensis* to antioxidant treatment with NAC, a thiol-reducing agent commonly used in clinical protocols, for five consecutive days before euthanasia. We first analysed parameters of oxidative stress in order to determine if NAC treatment was effective in reducing oxidative damage induced by *L. amazonensis* infection. Infected animals presented different alterations in SOD1 (Cu/Zn) and SOD2 (Mn) protein content; while SOD1 was decreased, SOD2 was increased. Nonetheless, NAC had no effect on these changes (Fig. 3A-B). On the other hand, the total content of sulfhydryl groups (Fig. 3C) were enhanced compared to infected group, suggesting an antioxidant effect. Nitrotyrosine levels (Fig. 3D) were significantly reduced in infected animals treated with NAC. These results altogether indicate that NAC treatment did not affect the adaptive modulation in the levels of SOD1 and two isoforms but exerted a protective effect against oxidative damage to proteins occurring in infected animals.

**Fig. 4:**
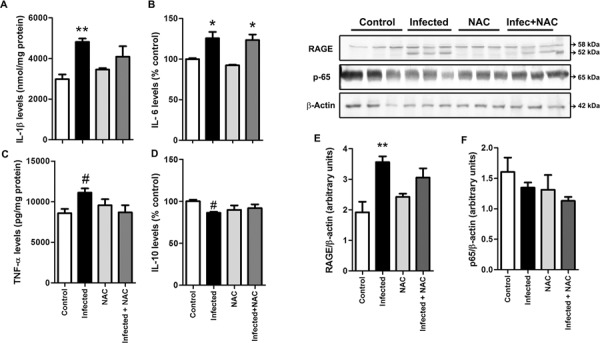
: effect of *N*-acetyl-cysteine (NAC) on molecular markers of pro-inflammatory activation in liver. Animals infected with *Leishmania amazonensis* were subjected to five consecutive days of NAC (NAC, 20 mg/kg b.w.) i.p. injection before euthanasia and tissue collection. Tissue levels of IL-1β (A), IL-6 (B), TNF-α (C) and IL-10 (D) were assessed by ELISA. The content of RAGE (E) and the total fraction of NF-kB subunit p65 (F) were assessed by western blot (representative blots depicted above graphs; b-actin was used as internal marker control). Values represent mean ± standard error of the mean (SEM) of samples obtained from five mice of two distinct groups (two different infections). Data were analysed by ANOVA. Bonferroni’s multiple comparison test was applied to confirm efficacy of experiment (control vs. infected groups, #p < 0.05). Tukey’s multiple comparison was applied to detect significant differences between all groups; *p < 0.05 and **p < 0.0001 compared to control group. No significant differences (p < 0.05) were detected between “infected” and “infected + NAC” groups.

Next, we analysed the effect of antioxidant treatment on parameters of pro-inflammatory activation in the liver of infected animals. The pro-inflammatory cytokines IL-1β (Fig. 4A), IL-6 (Fig. 4B) and TNF-α (Fig. 4C) were increased while the anti-inflammatory cytokine IL-10 was decreased (Fig. 4D) in the liver of infected animals compared to control group. Treatment with NAC did not significantly affect liver cytokine levels (Fig. 4A-D). We also analysed the immunocontent of the receptor for advanced glycation endproducts (RAGE), a receptor commonly associated to chronic pro-inflammatory states through NADPH oxidase-dependent superoxide production and NF-kB activation (Yamamoto et al. 2011, Kuhla et al. 2013). Infected animals presented an increase in RAGE levels, which was not affected by antioxidant treatment with NAC (Fig. 4E). Besides, the levels of the NF-kB activator subunit p65 (total fraction - cytosolic and nuclear) were not altered in any group (Fig. 4F). These results, altogether, indicate that oxidative stress is not involved in the up-regulation of pro-inflammatory parameters observed in the liver of animals infected with *L. amazonesis*.

## DISCUSSION

*L. amazonensis* is the causative agent of cutaneous and cutaneous diffuse leishmaniasis. These forms of infection are characterised by the appearance of chronic lesions disseminated through the skin, constituting a disabling disease with difficult treatment. *Leishmania* parasite resides within macrophages and can cause inhibition of mitochondrial respiratory activity, inactivation of peroxidases, increased susceptibility to oxidant damage, inhibition of glycolysis, S-nitrosylation, ADP-ribosylation, tyrosine nitration of proteins, disruption of Fe-S clusters, zinc fingers or heme groups and peroxidation of membrane lipids (Mauel & Ransijn 1997). The therapies available for leishmaniasis have serious side effects causing high toxicity if using at long periods (de Menezes et al. 2015, Kaplum et al. 2016). Currently there is no vaccine available for prevention of *Leishmania* parasites for human use.

We investigated parameters of oxidative stress at systemic level, by analysing the effect of a subcutaneous infection in liver, kidney, spleen, heart and lung tissues, and found significant alterations only in liver. Oxidative stress in clinical cases and experimental models of leishmaniasis is generally analysed in the context of infected cells or focusing on the microenvironment (tissue) of the injury. We observed here that reduced thiol groups and nitrotyrosine levels of liver were altered towards a pro-oxidant state. Such modifications favour the formation of intra- and intermolecular cross-links of proteins, which in turn lead to conformational changes increasing hydrophobicity and aggregation. As a result, protein aggregates accumulate, inducing generalised cellular dysfunction (Mattson & Magnus 2006).

We observed that SOD activity is enhanced and the non-enzymatic antioxidant capacity is decreased, indicating a state of oxidative stress. Modifications in overall antioxidant capacity of cells/tissues (i.e., the sum of non-enzymatic and enzymatic systems) may take place in response either to availability of dietary antioxidants (such as ascorbate or other exogenous antioxidants obtained in the diet), synthesis of endogenous antioxidants (such as glutathione, uric acid and bilirrubin) or modulation of antioxidant enzymes (Rahman 2007). Immunodetection of SOD1 and two isoforms demonstrated that SOD isoforms respond to *L. amazonensis* infection in different manners, although overall activity is enhanced. Up-regulation of SOD activity and protein content is a common response to superoxide production, which may be triggered by enhanced electron transfer chain in mitochondria or NADPH oxidase activation in cytosol (Baptista et al. 2012).

Recently study conducted with mouse peritoneal macrophages demonstrated that some consequences caused by *Leishmania* infection is probably mediated by an increase of reactive oxygen species, inducing cell death by apoptosis (da Silva et al. 2016). In addition the increases in reactive oxygen species might be regulating the inflammation caused in *L. amazonensis* infection (Roma et al. 2016).

The infection of BALB⁄c mice with *L. amazonensis* causes the development of polarised Th1 or Th2 responses, where a Th2 response results in increased number of parasites and tissue injury (Guerra et al. 2010). In different conditions, liver inflammation is associated to increased production of oxidants, thus resulting in cellular damage and progression of hepatic fibrosis (Muriel 2009). To evaluate if the redox imbalance caused *L. amazonensis* infection in liver was associated to the modulation of inflammatory mediators, we tested the effect of an acute administration of the antioxidant NAC on the levels of different molecular pro-inflammatory markers. NAC was effective in inhibiting nitrotyrosine formation and sulfhydryl oxidation, but it did not significantly affect changes in tissue IL-1β, IL-6, TNF-α and IL-10 (anti-inflammatory) content. Here, it is important to note that although mean values for IL-1β and TNF-α seem decreased in the group of infected animals receiving NAC, no statistically significant differences were pointed by ANOVA, which takes into account the variations in mean values of all groups in the experiments. We may not discharge the possibility that a more prolonged treatment with NAC could produce a more consistent effect in such cytokines, resulting in a decreased variance of the mean values observed in the groups that received NAC.

Our data were obtained four months after parasite infection, so it is possible that they may be associated to a chronic state. It is known that TNF-α promotes the induction of IL-6 and IL-1β; high levels of these cytokines can cause systemic inflammation (Rocha-Vieira et al. 2003) and the combination of oxidative stress and inflammatory activation is commonly associated to several degenerative processes triggered by parasitosis. Inflammatory target proteins, such as matrix metalloproteinase-9 (MMP-9) and cyclooxygenase-2 (COX-2) are associated with NADPH oxidase activation and reactive species overproduction in response to pro-inflammatory mediators, including TNF-α and IL-1b. However, in a parasitosis, these processes may be more strongly associated to either acute phases of infection or later stages of the chronic disease (de Oliveira et al. 2013). Besides, other inflammatory markers are expected to be increased in chronic conditions, such as RAGE.

RAGE has been identified as a central signal transduction receptor, mediating long-lasting NF-κB activation in various cell types. There is evidence for the involvement of RAGE in many liver diseases, including conditions with a well-defined chronic pro-inflammatory component (Zeng et al. 2004). RAGE modulates hepatic ischaemia-reperfusion injury by activation of a signal cascade linked to pro-inflammatory and cell death-promoting responses (Guo et al. 2008). It has also been reported that expression of this receptor is upregulated in many organ lesions, emphasising the pathophysiological role of this receptor in inflammation (Zeng et al. 2004). RAGE activation is directly related with oxidative stress and citotoxicity in several tissues, while increased RAGE expression is strongly associated to perpetuation of chronic sub-inflammatory states. We observed here that RAGE protein content was increased in the liver of infected animals, which strongly suggests up-regulation RAGE-dependent responses in this tissue. However, antioxidant treatment also had no significant effect on RAGE and this suggests that oxidative stress is not playing a role in the modulation of RAGE either. Besides, the immunocontent of the total fraction of NF-κB subunit p65 was not altered in any group. NF-κB-mediated transcriptional activation occurs independently of variations in p65 protein levels, which is consistent to a chronic inflammatory response (Calegari-Silva et al. 2009), so we may not rule out that NF-κB is inactive in the liver of infected animals based solely on these results.

The presence of proteins containing nitrotyrosine in the cutaneous lesions of BALB/c mice infected with *L. amazonensis* is already documented (Giorgio et al. 1996, 1998). We observed a significantly increased formation of nitrotyrosine in liver. Protein nitration is an important marker due to the potential ability to indicate acute and chronic disease states and predict disease risk (Linares et al. 2008). Increased nitrotyrosine is evidence for the formation of nitrating agents derived from nitric oxide (NO), such as peroxynitrite. Peroxynitrite is a potent oxidant agent that may be produced from the reaction of NO with superoxide radicals. Our data on SOD activity and protein content indicated a state of overproduction of superoxide radical in the liver of infected animals, and NO may be formed by diverse mechanisms in the course of infection. Protein tyrosine nitration occurs under basal physiological conditions and is several fold enhanced under circumstances that lead to augmented rates of oxidants and NO formation (Radi 2013). In the case of infectious diseases, the occurrence of protein tyrosine nitration in infected tissues and cells has been taken as evidence for the involvement of NO-derived oxidants in phagocyte microbicidal mechanisms.

Redox reactions involving sulfhydyl groups are very important in liver metabolism. This tissue plays a major role in detoxification of a wide variety of substances, from either exogenous or endogenous sources (Aparicio-Bautista et al. 2013). Sulfhydyl groups from glutathione (GSH) and other thiol-containing proteins in liver serve as a systemic redox buffer, providing electrons to reduce the excess of electrophiles that otherwise would cause damage to biomolecules. GSH is used in conjugation reactions for detoxification of electrophilic xenobiotics and endogenous metabolic products, one of the major roles exerted by liver. Besides, GSH and thiol-containing proteins are up-regulated in response to oxidative stress, as their reduced sulfhydryl groups are able to scavenge superoxide and hydroxyl radicals (Krinsky & Johnson 2005). Here, we observed that the liver of infected animals presented a decreased content of free (reduced) sulfhydryl groups, and NAC treatment was able to recover these levels. This suggests that hepatic functions dependent on the availability of reduced thiol groups in liver may be compromised at some stage of infection caused by *L. amazonensis*. Chronic depletion of free sulfhydryl groups and oxidative damage in liver are associated to liver steatosis and necrosis, while the association with inflammation evolves to tissue fibrosis and cirrhosis (Chen et al. 2013). Thus, although NAC treatment did not affect the up-regulation in pro-inflammatory markers in the liver of infected animals, a therapeutic application could contribute to preserve liver function, as it was effective in decreasing oxidative stress. In previous works, NAC was demonstrated to exert protective effects in liver in animals subjected to hypercholesterolemic diet, at much higher doses than we used here (230 mg/kg) (Korou et al. 2010). Besides, NAC at 200 mg/kg was administered to ApoE ^-/-^ mice prevented the acceleration of atherosclerosis in this model (Ivanovski et al. 2005). In these and other works, NAC at doses above 200 mg/kg did not show toxicity and were chosen based on previous toxicity assessments. It is possible that with more elevated doses of NAC we could have observed effects on inflammatory parameters as well.

In conclusion, we demonstrated here that BALB/c mice subjected to infection with *L. amazonensis* present altered markers of oxidative stress in liver; these include increased SOD activity, decrease on levels of free sulfhydryl groups, and non-enzymatic antioxidant capacity, enhanced nitrotyrosine levels, decreased SOD1 levels and increased SOD2 levels. Acute treatment of five daily injections with NAC decreased liver oxidative stress parameters, but it did not affect the levels of molecular markers of pro-inflammatory activation, such as the IL-1β, IL-6, TNF-α, IL-10 and RAGE. Nonetheless, our results suggest that the clinical use of NAC could be added to available therapies for leishmaniasis, which could help to prevent the development of liver disorders resulting from the association between oxidative stress and liver inflammation.

## References

[B1] Alvar J, Vélez ID, Bern C, Herrero M, Desjeux P, Cano J (2012). Leishmaniasis worldwide and global estimates of its incidence. PLoS ONE.

[B2] Aparicio-Bautista DI, Perez-Carreon JI, Gutierrez-Najera N, Reyes-Grajeda JP, Arellanes-Robledo J, Vasquez-Garzon VR (2013). Comparative proteomic analysis of thiol proteins in the liver after oxidative stress induced by diethylnitrosamine. Biochim Biophys Acta.

[B3] Baptista G, Dupuy AM, Jaussent A, Durant R, Ventura E, Sauguet P (2012). Low-grade chronic inflammation and superoxide anion production by NADPH oxidase are the main determinants of physical frailty in older adults. Free Radic Res.

[B4] Bisti S, Konidou G, Boelaert J, Lebastard M, Soteriadou K (2006). The prevention of the growth of Leishmania major progeny in BALB/c iron-loaded mice: a process coupled to increased oxidative burst, the amplitude and duration of which depend on initial parasite developmental stage and dose. Microbes Infect.

[B5] Blos M, Schleicher U, Rocha FJS, Meissner U, Rollinghoff M, Bogdan C (2003). Organ-specific and stage-dependent control of Leishmania major infection by inducible nitric oxide synthase and phagocyte NADPH oxidase. Eur J Immunol.

[B6] Bradford MM (1976). A rapid and sensitive method for the quantitation of microgram quantities of protein utilizing the principle of protein-dye binding. Anal Biochem.

[B7] Calegari-Silva TC, Pereira RM, De-Melo LD, Saraiva EM, Soares DC, Bellio M (2009). NF-kappaB-mediated repression of iNOS expression in Leishmania amazonensis macrophage infection. Immunol Lett.

[B8] Chen Y, Dong H, Thompson DC, Shertzer HG, Nebert DW, Vasiliou V (2013). Glutathione defense mechanism in liver injury: insights from animal models. Food Chem Toxicol.

[B9] da Silva BJ, da Silva RR, Rodrigues AP, Farias LH, do Nascimento JL, Silva EO (2016). Physalis angulata induces death of promastigotes and amastigotes of Leishmania (Leishmania) amazonensis via the generation of reactive oxygen species. Micron.

[B10] de Menezes JPB, Guedes CES, Petersen AL, Fraga DBM, Veras PST (2015). Advances in development of new treatment for leishmaniasis. Biomed Res Int.

[B11] de Oliveira RB, Senger MR, Vasques LM, Gasparotto J, dos Santos JP, Pasquali MA (2013). Schistosoma mansoni infection causes oxidative stress and alters receptor for advanced glycation endproduct (RAGE) and tau levels in multiple organs in mice. Int J Parasitol.

[B12] Desjeux P (2004). Leishmaniasis: current situation and new perspectives. Comp Immunol Microbiol Infect Dis.

[B13] di Penta A, Moreno B, Reix S, Fernandez-Diez B, Villanueva M, Errea O (2013). Oxidative stress and proinflammatory cytokines contribute to demyelination and axonal damage in a cerebellar culture model of neuroinflammation. PLoS ONE.

[B14] Giorgio S, Linares E, Capurro ML, de Bianchi AG, Augusto O (1996). Formation of nitrosyl hemoglobin and nitrotyrosine during murine leishmaniasis. Photochem Photobiol.

[B15] Giorgio S, Linares E, Ischiropoulos H, Von Zuben FJ, Yamada A, Augusto O (1998). In vivo formation of electron paramagnetic resonance-detectable nitric oxide and of nitrotyrosine is not impaired during murine leishmaniasis. Infect Immun.

[B16] Guerra CS, Silva RM, Carvalho LO, Calabrese KS, Bozza PT, Corte-Real S (2010). Histopathological analysis of initial cellular response in TLR-2 deficient mice experimentally infected by Leishmania (L.) amazonensis. Int J Exp Pathol.

[B17] Guo ZJ, Niu HX, Hou FF, Zhang L, Fu N, Nagai R (2008). Advanced oxidation protein products activate vascular endothelial cells via a RAGE-mediated signaling pathway. Antioxid Redox Signal.

[B18] Halliwell B, Gutteridge JMC (2007). Free radicals in biology and medicine.

[B19] Ivanovski O, Szumilak D, Nguyen-Khoa T, Ruellan N, Phan O, Lacour B (2005). The antioxidant N-acetylcysteine prevents accelerated atherosclerosis in uremic apolipoprotein E knockout mice. Kidney Int.

[B20] Kaplum V, Cogo J, Sangi DP, Ueda-Nakamura T, Correa AG, Nakamura CV (2016). In vitro and in vivo activities of 2,3-diarylsubstituted quinoxaline derivatives against Leishmania amazonensis. Antimicrob Agents Chemother.

[B21] Korou LM, Agrogiannis G, Pantopoulou A, Vlachos IS, Iliopoulos D, Karatzas T (2010). Comparative antilipidemic effect of N-acetylcysteine and sesame oil administration in diet-induced hypercholesterolemic mice. Lipids Health Dis.

[B22] Krinsky IN, Johnson JE (2005). Carotenoid actions and their relation to health and disease. Mol Aspects Med.

[B23] Kuhla A, Norden J, Abshagen K, Menger MD, Vollmar B (2013). RAGE blockade and hepatic microcirculation in experimental endotoxaemic liver failure. Br J Surg.

[B24] Linares E, Giorgio S, Augusto O (2008). Inhibition of in vivo leishmanicidal mechanisms by tempol: nitric oxide down-regulation and oxidant scavenging. Free Radic Biol Med.

[B25] Mattson MP, Magnus T (2006). Ageing and neuronal vulnerability. Nat Rev Neurosci.

[B26] Mauel J, Ransijn A (1997). Leishmania spp.: mechanisms of toxicity of nitrogen oxidation products. Exp Parasitol.

[B27] Muriel P (2009). Role of free radicals in liver diseases. Hepatol Int.

[B28] Radi R (2013). Protein tyrosine nitration: biochemical mechanisms and structural basis of functional effects. Acc Chem Res.

[B29] Rahman K (2007). Studies on free radicals, antioxidants, and co-factors. Clin Interv Aging.

[B30] Rocha-Vieira E, Ferreira E, Vianna P, de Faria DR, Gaze ST, Dutra WO (2003). Histopathological outcome of Leishmania major-infected BALB/c mice is improved by oral treatment with N-acetyl-l-cysteine. Immunology.

[B31] Roma EH, Macedo JP, Goes GR, Gonçalves JL, Castro W, Cisalpino D (2016). Impact of reactive oxygen species (ROS) on the control of parasite loads and inflammation in Leishmania amazonensis infection. Parasit Vectors.

[B32] Yamamoto Y, Harashima A, Saito H, Tsuneyama K, Munesue S, Motoyoshi S (2011). Septic shock is associated with receptor for advanced glycation end products ligation of LPS. J Immunol.

[B33] Zeng S, Feirt N, Goldstein M, Guarrera J, Ippagunta N, Ekong U (2004). Blockade of receptor for advanced glycation end product (RAGE) attenuates ischemia and reperfusion injury to the liver in mice. Hepatology.

